# Brain Atrophy Is Associated with Hematoma Expansion in Intracerebral Hemorrhage, Depending on Coagulation Status

**DOI:** 10.3390/jcm14072227

**Published:** 2025-03-25

**Authors:** Anna Speth, Andrea Dell’Orco, Justus F. Kleine, Christopher Güttler, Andrea Morotti, Horst Urbach, Georg Bohner, Michael Scheel, Jawed Nawabi, Frieder Schlunk

**Affiliations:** 1 Department of Neuroradiology, Charité—Universitätsmedizin Berlin, Corporate Member of Freie Universität Berlin, Humboldt—Universität zu Berlin, and Berlin Institute of Health, 14197 Berlin, Germanyandrea.dellorco@charite.de (A.D.); justus.kleine@charite.de (J.F.K.); christopher.guettler@charite.de (C.G.); georg.bohner@charite.de (G.B.); michael.scheel@charite.de (M.S.); jawed.nawabi@charite.de (J.N.); 2 Neurology Unit, Department of Neurological Sciences and Vision, ASST Spedali Civili, 25123 Brescia, Italy; andrea.morotti85@gmail.com; 3 Department of Neuroradiology, Medical Center-University of Freiburg, Breisacher Strasse 64, 79106 Freiburg im Breisgau, Germany; horst.urbach@uniklinik-freiburg.de

**Keywords:** intracerebral hemorrhage, anticoagulation, brain atrophy, computed tomography, hematoma expansion

## Abstract

**Background/Objectives:** This study aimed to research the potential association between brain atrophy and hematoma expansion (HE) in intracerebral hemorrhage (ICH). **Methods:** A retrospective analysis was conducted using data from patients with primary ICH in our stroke database. ICH volumes from initial and follow-up CT scans were manually segmented. Total brain and intracranial volumes were quantified using an automated head CT segmentation method. Normalized brain volume (NBV) was calculated by dividing the total brain volume by the total intracranial volume to account for individual head size differences. The relationship between the NBV and hematoma expansion was assessed using linear regression, adjusting for other variables influencing hematoma expansion. **Results:** Our final analysis included 420 patients. Brain atrophy (lower NBV) was associated with hematoma growth (>0 mL) in patients not on oral anticoagulants (β = −0.159, *p* = 0.032). A strong association was observed in patients using vitamin K antagonists (β = −0.667, *p* = 0.006) but not in those on direct oral anticoagulants (DOACs; (β = −0.159, *p* = 0.436)). Results remained significant in patients not on oral anticoagulants and in those on VKAs when hematoma expansion was defined as a volume increase >6 mL or >33%. **Conclusions:** This research provides initial evidence that brain atrophy is a risk factor for hematoma expansion, depending on the patient’s coagulation status. These findings could enhance risk stratification for acute clinical management and deepen understanding of the biological mechanisms behind hematoma expansion.

## 1. Introduction

Enhancing the clinical management of patients with intracerebral hemorrhage (ICH) is critically important. Roughly one-third of these patients undergo hematoma expansion (HE) after hospital admission, which is the most significant prognostic factor affecting their clinical outcomes [1]. While they offer a potential target for treatment, the biological mechanisms behind hematoma growth are still not well-understood and are likely a complex interplay of facilitating and inhibiting factors. In the 1970s, Fisher introduced the theory that the force of a growing hematoma causes secondary vessel rupture and bleeding by shearing and tearing [2,3]. This avalanche concept was recently confirmed with a novel mouse model, which demonstrated that secondary bleeding occurs depending on the speed and volume of the initial hematoma growth [4]. Cessation of bleeding is achieved presumably by coagulation activation and increasing the counter pressure of the surrounding tissue [5]. Decreased counter pressure and subsequent hematoma expansion are well-known complications after a decompressive craniectomy [6,7]. However, what occurs when the counter pressure is reduced, as it must be in the case of patients with pronounced brain atrophy, is unknown. Intuitively, one would assume that bleeding is facilitated due to a decreased ratio between solid tissues and fluids since tissues can be shifted more easily to other compartments. Furthermore, this effect might differ depending on anatomical locations, since the bleed will more or less be in proximity to the subarachnoid spaces. This could be one factor explaining the differences in hematoma expansion between deep and lobar intracerebral hemorrhages. Indeed, lobar hemorrhages were found to be larger than those located deeply in the brain [8,9].

A well-known factor that aggravates hematoma expansion is oral anticoagulation therapy with vitamin K antagonists (VKAs) or direct oral anticoagulants (DOACs) [10,11,12]. The coagulation system and the tamponade effect presumably support each other. If clotting is impaired, the tamponade effect may be reduced or ineffective. Since loss of brain volume and oral anticoagulation therapy often coincide in the elderly, investigating a possible interaction is of clinical importance. However, because the mechanisms of action of VKAs and DOACs are fundamentally different, these must be considered separately. VKAs exert their effects by inhibiting clotting factors II, VII, IX, and X, while DOACs directly inhibit thrombin (IIa) or factor Xa. Their interplay with the tamponade effect may, therefore, vary.

Progressive brain atrophy is common in normal aging. While the peak brain volume is achieved relatively early in life, cerebral atrophy increases after the age of 65 and is accelerated in patients with neurodegenerative diseases or following traumatic brain injury or cerebrovascular disease [13,14]. Interestingly, in aging patients, there is a disproportionally greater loss of grey matter tissue compared to white matter or subcortical volumes [15]. As neuronal cell loss occurs, there is an accompanying increase in cerebrospinal fluid levels. It is imaginable that a growing mass, such as an intracerebral hemorrhage, follows a different growth pattern if there is less tissue resistance in its proximity.

In this study, we retrospectively assessed the hypothesis that brain atrophy is associated with increased hematoma expansion in ICH.

## 2. Methods

### 2.1. Study Population

The data that support the findings of this study are available from the corresponding author upon reasonable request.

We identified all patients from January 2014 to July 2019 with a diagnosis of ICH in our prospectively maintained stroke database (Charité Universitätsmedizin Berlin, Berlin, Germany). Patient baseline characteristics, encompassing Glasgow Coma Scale (GCS) scores upon admission and modified Rankin Scale (mRS) scores at discharge, were extracted from medical records. Additionally, data regarding diabetes mellitus, blood pressure parameters, and usage of oral anticoagulation and antiplatelet medications. In addition, details of follow-up procedures, such as craniectomies or the placement of intraventricular drainage, were sourced from patients’ clinical records and follow-up CT scans. A dichotomized neurological outcome was determined based on the modified Rankin Scale (mRS), with a score of ≤2 indicating a favorable outcome and a score of >2 indicating an unfavorable outcome. Patients >18 years with a spontaneous intracerebral hemorrhage (supratentorial or infratentorial) were included if they had initial and follow-up non-contrast computed tomography (NCCT) scans. Referrals from external hospitals were also included if the initial NCCT scan was available. Patients undergoing oral anticoagulant treatment were included. Exclusion criteria were underlying brain tumors, vascular malformations, traumatic intracranial hemorrhages, primary intraventricular hemorrhages, or secondary ICHs from hemorrhagic transformation of ischemic infarctions. Patients were excluded if they underwent surgical procedures before their follow-up CT scan (e.g., a decompressive craniectomy or hematoma evacuation) to avoid inaccurate measurements of hematoma volume. However, patients who had surgery after their follow-up CT scan were included in this study.

This retrospective study received approval from the ethics committee (Ethik-Komission der Charité Berlin; identification number EA4/009/20). In line with relevant institutional guidelines, informed patient consent was waived due to the use of anonymized data in this retrospective study. This study was conducted in accordance with the declaration of Helsinki.

### 2.2. Image Acquisition

Non-contrast CT scans were conducted at Charité University Hospital (Berlin, Germany) using an 80- or 320-slice Toshiba Aquilion Prime scanner. The imaging parameters were set as follows: incremental acquisition at 120 kV and 280 mA, with 1.0 mm and 5 mm slice reconstructions. All datasets were reviewed for quality, and those with severe motion artifacts were excluded.

### 2.3. Image Analysis—ICH Volume

An experienced neuroradiologist confirmed and assessed the location of the hematoma and the presence of any intraventricular hemorrhage. The ICH was classified as deep (basal ganglia and thalamic), lobar, brain stem, or cerebellum. Hematoma volumes were measured on axial slices (slice thickness of 5 mm) using an open-source semi-automated segmentation tool (ITK-snap version 3.8.0) by different readers experienced with stroke imaging. All raters were blinded to all demographic and outcome data and measured hematoma volumes on CT scans in a random order. The readers were not engaged in the clinical care of the patients included in this study.

### 2.4. Image Analysis—Brain Volume

**Figure 1:**
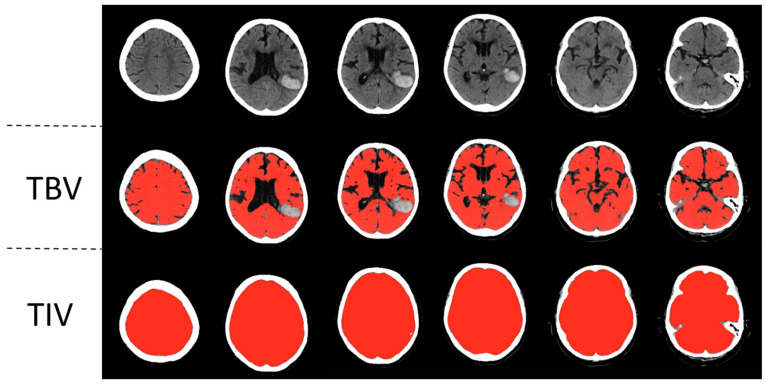
Automatic segmentation of a left parietotemporal lobar intracerebral hemorrhage. Segmentations of total brain volume (TBV) and total intracranial volume (TIV) are in the second and third rows, respectively.

To measure the total brain volume and the total intracranial volume, a previously published automated head CT segmentation method (CTseg: https://github.com/NuroAI/CTSeg, accessed on 1 September 2020), was used (see Figure 1). This method has been previously published elsewhere [16]. Briefly, it employs the adapted unified segmentation algorithm from SPM Toolbox, Version 12, and incorporates a CT template to initiate the affine registration process.

SPM 12 itself is a broadly applied algorithm that is used mostly for the MRI segmentation of different parts of the brain, e.g., gray matter, white matter, or cerebral spinal fluid. First, the likelihood of each voxel belonging to a specific structure is calculated. This generates a probabilistic map. It gradually modulates the intensity distribution of the various brain tissues and then calculates the probability for each tissue using the Bayes rule. Next, spatial normalization is performed by adapting the standard tissue probability map to the calculated probabilistic map. This method is independent of the absolute tissue intensity of the original image. Thus, this method is applicable to different modalities. Here, CTSeg applies this algorithm to CT images. For this purpose, a CT template was used, which was compared with a standardized MR imaging template. As a first step, the voxel intensities of the CT image are adjusted to match the intensity space of the CT template. Secondly, The CT image is aligned with the CT template to secure the correct spatial orientation. Then, the registered CT image is segmented using SPM to create tissue probability maps for the different tissues. These maps represent the probability that a certain region of the image belongs to a specific type of tissue. Afterward, the probability maps are transformed back to the original space of the CT image. Finally, the probability maps are converted into binary maps by applying optimal threshold values. If a specific pixel surpasses this specific threshold, it is finally labeled as a specific tissue. This is how the final segmentation map is generated [16,17].

This method has shown excellent agreement with manually segmented head CTs for both intracranial volume and total brain volume in the past [16]. CTseg has already been successfully applied in different studies. For example, it was used to describe how brain atrophy increases in septic ICU patients [18]. In another study, it was applied to show how brain atrophy affects the effect of endovascular therapy in patients with acute ischemic stroke due to large vessel occlusion [19]. To validate this method, we performed an additional analysis on 23 randomly selected consecutive head CT scans, which were independently assessed by an experienced neuroradiologist using the GCA visual atrophy score adapted for total brain volume. The results showed excellent agreement with the automated brain volume measurements (rs = −0.87369, *p* < 0.001) using Spearman’s rank correlation test.

After measurements, all segmentations were reviewed by an experienced neuroradiologist, and incorrect measurements (e.g., incomplete segmentations) were excluded.

### 2.5. Statistical Analysis

Statistical analysis was performed with R v3.6.1 and the libraries ggplot2 v3.1.1 and tidyverse v1.2.1. For normally distributed data, the mean and standard deviations (SDs) were utilized, while for non-normally distributed data, the median and range were employed, respectively. Categorical variables are reported as counts and percentages. A *p*-value of less than 0.05 was considered statistically significant. The relationships between the NBV and HE were tested using two different definitions. HE was either defined as an absolute increase in volume (HE > 0 mL) or an increase in volume >6 mL or >33% between the baseline CT and the follow-up CT (HE > 6 mL or >33%) [20]. Relationships between the NBV and hematoma expansion were measured with linear regression. Initial hematoma volume, age, sex, hypertension, diabetes, and use of anticoagulants or antiplatelets were considered as covariates. The association of the NBV with HE was first tested on the whole cohort, then for the anticoagulant subgroups, and finally for the different locations. Results are reported as standardized β.

## 3. Results

### 3.1. Study Participants and CT Data

**Figure 2:**
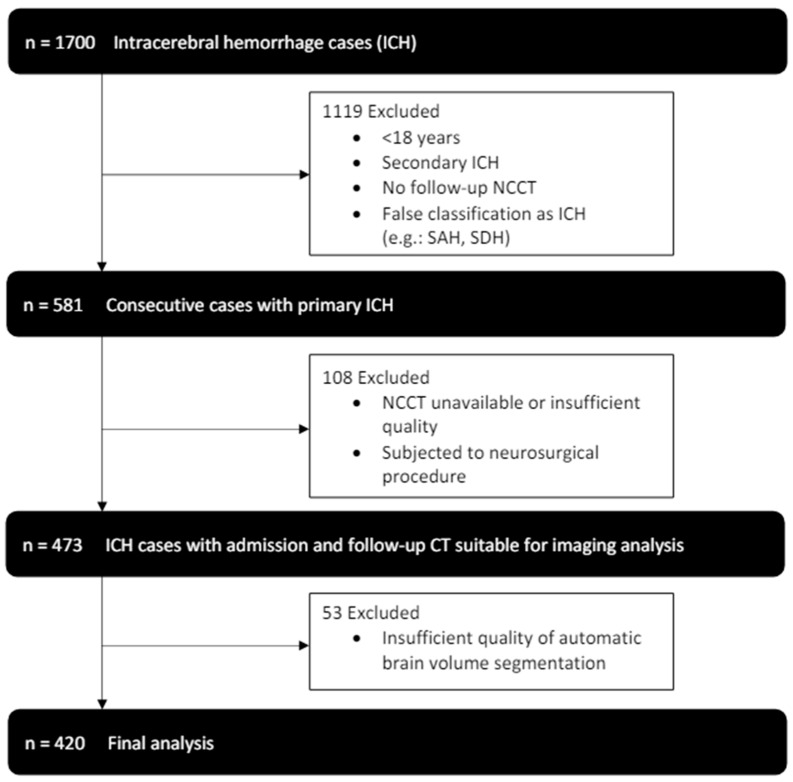
Enrollment flow chart and study inclusion and exclusion criteria.

From January 2014 to July 2019, 1700 patients were identified in our database with the diagnosis of intracerebral hemorrhage. After application of the exclusion criteria, 420 patients, who received an initial NCCT scan as well as follow-up imaging were included in our final analysis (see Figure 2). Patients had a median age of 72 (61–79) and 184 (44%) were female. Histories of hypertension (345 (82%)) and diabetes (70 (17%)) were frequent (see Table 1 for further baseline demographics and clinical characteristics).

The location of the ICH was deep hemispheric in 198 (47%), lobar in 160 (38%), and infratentorial (brainstem or cerebellar) in 60 subjects (14%). The mean hematoma volume at admission was 19 (7–41) mL and 23 (8–46) mL at follow-up. The median time from symptom onset to the admission CT scan was 135 (73–459) min, and the median time from the admission CT scan to the first follow-up scan was 975 (365–1525) min. For anticoagulated patients, the median time from symptom onset to the admission CT scan was 139 (84–374) min, with a median time of 899 (368–1496) minutes from the admission CT scan to the first follow-up scan. In non-anticoagulated patients, these times were 129 (67–463) min and 981 (341–1526) min, respectively. The mean NBV (total brain volume/total intracranial volume) was 0.871 ± 0.030. As expected, there was a correlation between NBV and patient age, with higher NBV values in younger individuals (*p* < 0.001).

### 3.2. NBV and Hematoma Growth

**Figure 3:**
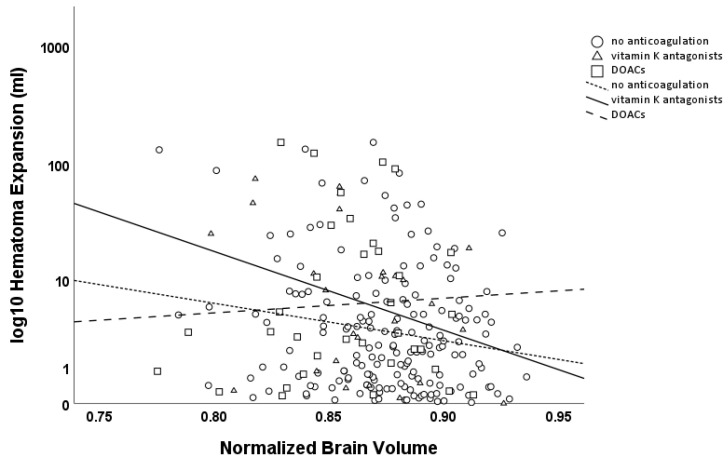
Scatter plot of hematoma expansion (logarithmic scale, base 10; mL) versus Normalized Brain Volume (NBV). Circles and the dashed line (with short dashes) represent patients without anticoagulation, while triangles and the solid line represent patients on VKAs, and squares with a dashed line (with longer dashes) represent patients on DOACs. Hematoma expansion was significantly associated with NBV in patients without anticoagulation therapy and in patients on VKAs, but not in patients on DOACs.

HE > 0 mL was negatively associated with the NBV (β = −0.093, *p* = 0.145), with a stronger effect for HE > 6 mL or >33% (β = −0.135, *p* = 0.127). Subgroup analysis revealed an association for both HE > 0 mL and HE > 6 mL or >33% in patients without anticoagulation therapy (HE > 0 mL β = −0.159, *p* = 0.032; HE > 6 mL or >33% β = −0.266, *p* = 0.043), indicating that brain atrophy was associated with the risk of further hematoma expansion. While no interaction was observed between brain atrophy and hematoma expansion in patients using oral anticoagulants as a whole, a strong association was found in the subgroup of patients on vitamin K antagonists (VKAs, HE > 0 mL β = −0.667, *p* = 0.006; HE > 6 mL or >33% β = −0.651, *p* = 0.025), while no such association was observed in those on direct oral anticoagulants (DOACs, HE > 0 mL β = −0.159, *p* = 0.436; HE > 6 mL or >33% β = −0.113, *p* = 0.607; see Figure 3).

Further analysis in the patient population not using oral anticoagulants revealed a stronger negative effect of the NBV in lobar ICH on HE > 0 mL (β = −0.226, *p* = 0.081), but not on HE > 6 mL or >33% (β = −0.140, *p* = 0.394). No effect for both HE > 0 mL and HE > 6 mL or >33% was seen in deep ICH (basal ganglia or thalamus; HE > 0 mL β = −0.053, *p* = 0.639; HE > 6 mL or >33% β = −0.152, *p* = 0.546) or in brainstem ICH (HE > 0 mL β = 0.018, *p* = 0.78; HE > 6 mL or >33% n = too low for statistical evaluation). A negative effect was also observed in cerebellar ICH for HE > 0; however, we believe that the number of cases included in our study is too low to draw a strong conclusion (β = −0.42, *p* = 0.1, n = 12; HE > 6 mL or >33% n = too low for statistical evaluation). See Table 2 and Table 3 for an overview of the data and the results for covariates.

### 3.3. NBV, Hematoma Growth, and Neurological Outcome

In the patient population, not using oral anticoagulants and a high modified ranking scale (mRS) at discharge (>2), we found a strong correlation between NBV and HE (HE > 0 mL β = −0.214, *p* = 0.014; HE > 6 mL or >33% β = −0.333 *p* = 0.022). No effect was seen in patients with a mRS ≤ 2 (HE > 0 mL β = 0.124, *p* = 0.7; HE > 6 mL or >33% n = too low for statistical evaluation).

## 4. Discussion

In this retrospective study, we investigated if brain atrophy is associated with hematoma expansion in ICH. Our data indeed show such an association, albeit dependent on the patient’s coagulation status. To our knowledge, this is the first study identifying brain atrophy as a risk factor for hematoma expansion in intracerebral hemorrhage.

It is conceivable that hematoma growth can decrease or stop when the counter pressure of the surrounding tissue, which increases in parallel with hematoma growth, overcomes the force of blood extravasating from the primary vessel [21]. Indeed, proximity to the hematoma’s center (e.g., where the forces of the bleeding source are stronger) was found to be a factor in increasing hematoma expansion along the surface of an intracerebral hemorrhage [22]. Recent experimental data suggest that there might be a threshold value for when local pressure from a fast-growing hematoma overcomes the counter pressure of the surrounding tissue, resulting in an avalanche of secondary vessel rupture and bleeding [4]. The present data suggest that this threshold may be lower in absolute terms with pronounced brain atrophy because of the presumably decreased tissue counter pressure. In comparison to deep hemorrhages, lobar hemorrhages might be more prone to such an effect because of their more superficial location and proximity to the subarachnoid spaces expanded by atrophy, which might explain the lack of effect of NBV in deep ICH. Other authors described that lobar ICH volumes on admission are bigger and that risk factors for deep and lobar ICHs differ from each other [23]. The relative contributions of mechanisms underlying hematoma expansion might therefore differ between different ICH locations.

In this study, the association of a lower total brain volume with hematoma expansion depended on coagulation status. We observed the strongest effect in patients using VKAs, while such an effect was absent in patients using DOACs. Consistent with our findings, experimental and human data reported smaller hematomas and better outcomes in DOAC-related intracerebral hemorrhages and traumatic intracranial hemorrhages as compared to VKAs [24,25,26,27]. Whereas VKAs affect coagulation by reducing the activity of factors II, VII, IX, and X in plasma, DOACs directly interfere with factor IIa (dabigatran) or factor Xa (rivaroxaban and apixaban). The direct inhibition of a single factor may enable alternative pathways in the clotting cascade, such as the direct activation of factor VIIa via factor III surrounding cerebral blood vessels [26]. While this provides a possible explanation for the reduced hematoma growth observed in patients using DOACs compared to VKAs, it does not explain why we observed an association between brain atrophy and hematoma expansion in non-anticoagulated patients. Another possibility is that specific patterns of brain atrophy may overlap with neurovascular diseases, such as cerebral amyloid angiopathy [28]. A larger amount of amyloid-β deposition may predispose to secondary vessel rupture and further hematoma growth, with the bleed following clusters of pre-damaged, more vulnerable vessels. We cannot exclude the possibility that specific patterns of brain atrophy were more prevalent in our cohort of non-anticoagulated patients compared to those on DOACs. Furthermore, neurodegenerative diseases such as Alzheimer’s disease are linked to inflammatory processes and impairment of the blood–brain barrier [29]. In ICHs, infiltration of inflammatory cells and blood–brain barrier disruption typically occur later in the course of acute intracerebral hemorrhage, after hematoma growth has usually ceased in most cases [30]. However, we cannot rule out the possibility that these markers of secondary brain injury could influence hematoma expansion, at least to some extent, if they are already present at the time of bleeding. In summary, our study is exploratory in nature and further research is needed to elucidate the pathophysiological mechanisms underlying our findings, which could be most effectively investigated in an experimental setting.

Sporadic publications have examined the influence of cerebral atrophy on clinical outcomes in patients with intracerebral hemorrhage. In two studies, brain atrophy was associated with worse outcomes after 90 days, which supports our findings. Presumably, this has to be attributed not only to hematoma expansion but also to other factors such as pre-existing neurodegenerative diseases, which are per se associated with worse neurological outcomes [31,32]. On the contrary, another study found a protective effect of brain atrophy on the 90-day outcome of patients with moderate volume basal ganglia hemorrhages [33]. While this finding does not contradict our results, the authors used the intercaudate distance and sylvian fissure ratio as parameters of linear brain atrophy, which might be less precise than the semiautomatic segmentation of the hematoma volume itself as applied in our study. In our study, brain atrophy was strongly associated with hematoma expansion in patients with a mRS > 2. In another study, no association between hematoma expansion and brain atrophy was found. A visual rating scale was used to grade brain atrophy in that context. Furthermore, the authors did not account for the hematoma’s location and the use of anticoagulants in their calculations. The study comprised a large number of non-lobar hemorrhages, which may be the reason for the divergent results [34].

Our findings have significant clinical implications. First, the identification of brain atrophy as a new risk factor for hematoma expansion in intracerebral hemorrhage introduces an important consideration for clinical guidelines and risk stratification scores in patients with acute ICH. This could potentially inform the development of more tailored and precise prediction models for hematoma expansion in the acute phase. Secondly, our data contribute to the growing body of knowledge regarding the mechanisms underlying hematoma growth, both with and without the background of anticoagulation. Understanding these mechanisms is crucial for the ultimate goal of developing more effective treatment options and improving patient outcomes.

Thirdly, our study suggests that strategies aimed at achieving hemostasis should perhaps not only focus on pharmacological agents that promote coagulation but also factor in the role of the described counter-pressure effect.

The strengths of our study include the large number of patients and the high data accuracy provided by the manual segmentation of intracerebral hemorrhage volumes on every admission and follow-up CT, which was reviewed by expert neuroradiologists, as well as the comprehensiveness of the clinical information collected for our study over a relatively large timeframe. Limitations include the retrospective study design. We cannot exclude a selection bias for our study since a large number of patients were excluded, mainly because of missing follow-up CT scans or false classifications as ICHs. We used unadjusted *p*-values since our analyses were prespecified and to avoid a loss in statistical power. We acknowledge that our results should be interpreted with caution and must be validated through subsequent studies.

## 5. Conclusions

Brain atrophy is associated with the expansion of intracerebral hematomas, depending on coagulation status, which should be taken into account in risk stratification for patients in the acute setting. A better understanding of the biological mechanisms behind hematoma expansion could ultimately lead to new randomized controlled trials and treatments limiting hematoma growth.

## Figures and Tables

**Table 1:** Comparison of baseline demographic, clinical and radiological characteristics in patients with acute intracerebral hemorrhage (ICH). NIHSS indicates National Institutes of Health Stroke Scale; mRS modified Rankin Scale; IQR interquartile range.

Variable	Total (n = 420)
Age, median (IQR), y	72 (61–79)
Female sex, No. (%)	184 (44)
Hypertension, No. (%)	345 (82)
Diabetes, No. (%)	70 (17)
Antiplatelet therapy, No. (%)	100 (24)
Anticoagulation therapy, No. (%)	123 (29)
- Vitamin K Antagonists (VKA), No. - Direct Oral Anticoagulants (DOAC), No. - Other Anticoag. (e.g., Heparin), No.	45 47 31
Glasgow Coma Scale score, median (IQR)	12 (8–14)
NIHSS admission, median (IQR)	10 (5–15)
mRS discharge, median (IQR)	5 (4–6)
Bleeding location, No. (%)	
- Deep - Lobar - Cerebellar - Brainstem	198 (47) 160 (38) 31 (7) 29 (7)
Intraventricular hemorrhage, No. (%)	181 (43)
Baseline ICH volume, median (IQR), mL	19 (7–41)
Follow ICH volume, median (IQR), mL	23 (8–46)
Time to admission CT scan, median (IQR), min	135 (73–459)
Time CT admission to follow up, median (IQR), min	975 (365–1525)

**Table 2:** Results of multivariable linear regression analysis of absolute hematoma growth (HE > 0 mL) between the baseline CT and the follow-up CT. Panels show non-anticoagulated patients (No OAT), patients on VKAs, and patients on DOACs. Significance levels: * *p* < 0.05, *** *p* < 0.001.

	No OAT		VKAs		DOACs	
Covariate	ß	*p* Value	ß	*p* Value	ß	*p* Value
Age	−0.135	0.1	−0.486	0.031 *	0.209	0.276
Sex (male)	−0.08	0.281	−0.063	0.771	0.095	0.627
NBV	−0.159	0.032 *	−0.667	0.006 *	−0.159	0.436
Hypertension	−0.055	0.454	−0.182	0.439	−0.371	0.082
Diabetes	−0.07	0.327	0.09	0.691	−0.444	0.041 *
Antiplatelet therapy	0.156	0.032 *	0.125	0.504	−0.014	0.952
Initial hematoma vol.	0.331	<0.001 ***	0.399	0.056	0.317	0.1

**Table 3:** Results of multivariable linear regression analysis in non-anticoagulated patients of absolute hematoma growth (HE > 0 mL) and hematoma expansion defined as an increase of volume > 6 mL or >33% between the baseline CT and the follow-up CT (HE > 6mL or >33%). Panels show deep vs. lobar intracerebral hemorrhage. Asterisks indicate statistical significance: * for *p* < 0.05, ** for *p* < 0.01.

	Intracerebral Hemorrhage							
	Deep				Lobar			
	HE > 0 mL		HE > 6 mL or 33%		HE > 0 mL		HE > 6 mL or 33%	
Covariate	ß	*p* Value	ß	*p* Value	ß	*p* Value	ß	*p* Value
Age	−0.085	0.49	−0.440	0.08	−0.289	0.049 *	−0.471	0.008 **
Sex (male)	−0.136	0.24	−0.267	0.27	−0.028	0.83	−0.176	0.35
NBV	−0.053	0.64	−0.152	0.55	−0.226	0.081	−0.14	0.39
Hypertension	0.094	0.39	0.024	0.92	−0.093	0.49	−0.295	0.07
Diabetes	−0.049	0.66	−0.115	0.64	−0.086	0.5	−0.22	0.18
Antiplatelet therapy	0.103	0.34	0.084	0.73	0.246	0.06	0.501	0.016 *
Initial hematoma vol.	0.286	0.007 **	−0.048	0.832	0.276	0.03 *	0.121	0.43

## Data Availability

The data that support the findings of this study are available from the corresponding author upon reasonable request.
